# Supplementary figures and images for: The efficacy of sampling strategies for estimating scabies prevalence
Source: PLoS Negl Trop Dis. 2022 Jun 9;16(6):e0010456. doi: 10.1371/journal.pntd.0010456 (PMC9216578; doi:10.1371/journal.pntd.0010456)

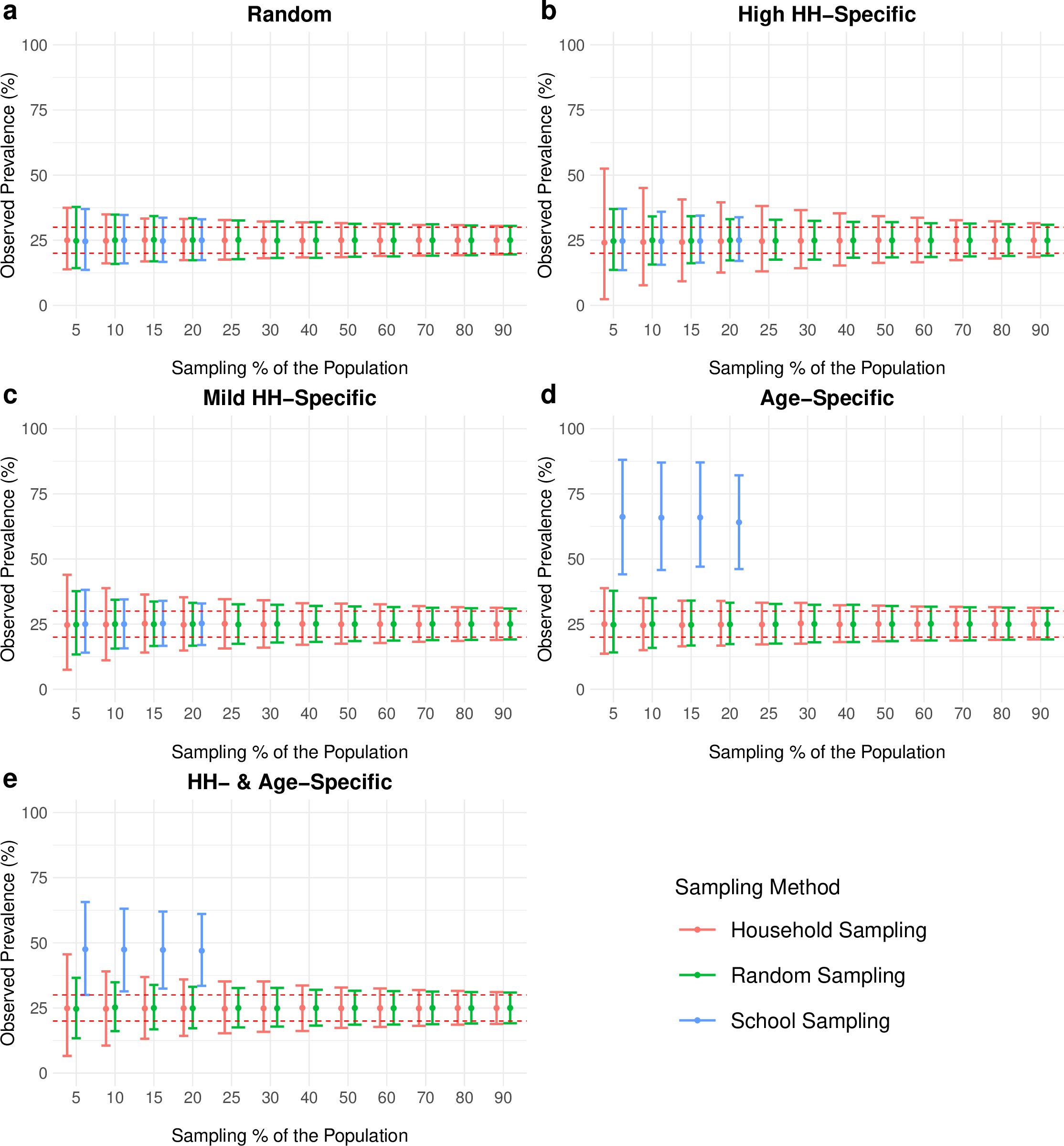

Supplement: S1 Fig — The results (median and 2.5% to 97.5% quantiles) are plotted for an exemplar input prevalence percentage between 20–30% across all population sizes where (a) random, (b) high household-specific, (c) mild household-specific, (d) age-specific, (e) age-and-household-specific scabies assignment method is used. Red dashed lines represent 20% and 30% prevalence. Error bars represent the 2.5% to 97.5% quantiles. (TIF) [file pntd.0010456.s001.tif]

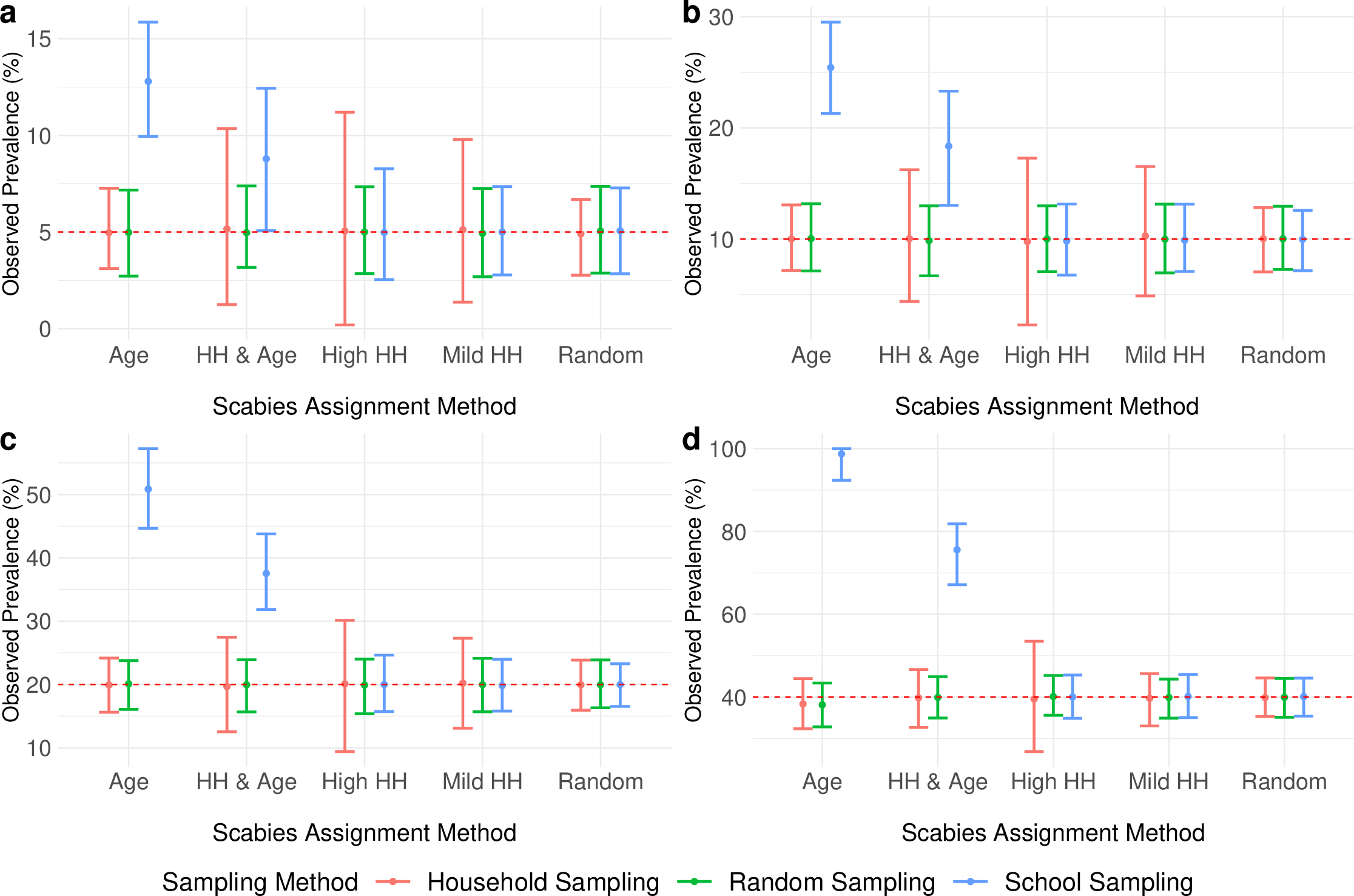

Supplement: S2 Fig — The results (median and 2.5% to 97.5% quantiles) are plotted for four exemplar input prevalence percentages of (a) 5%, (b) 10%, (c) 20%, (d) 40% across all population sizes with a sampling percentage of 20%. Red dashed lines represent the input prevalences. (TIF) [file pntd.0010456.s002.tif]

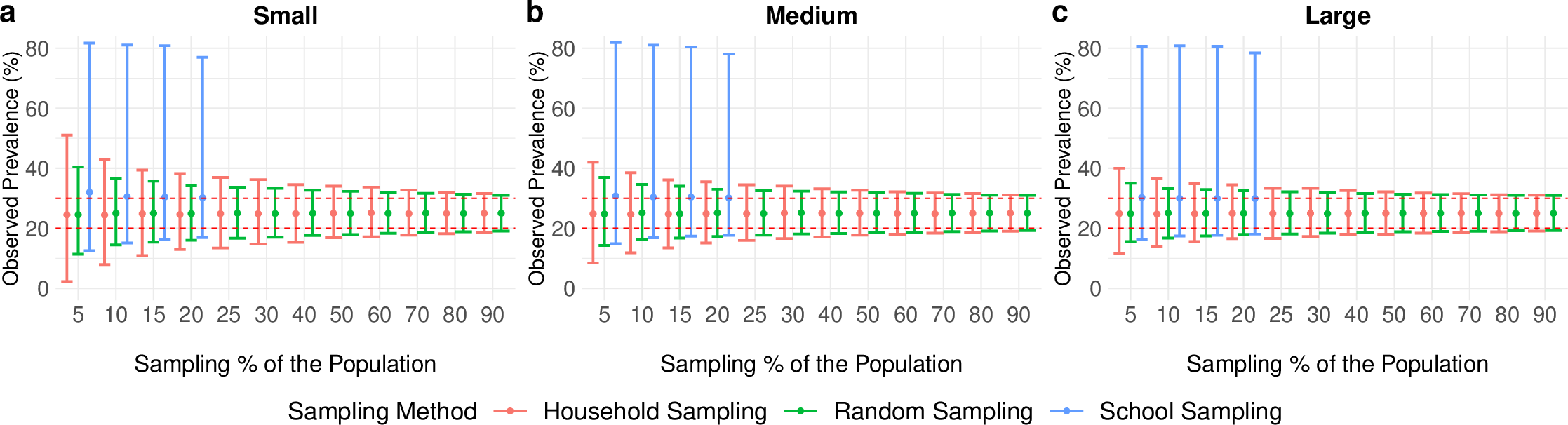

Supplement: S3 Fig — The results (median and 2.5% to 97.5% quantiles) are plotted for an exemplar input prevalence percentage between 20–30% with a sampling percentage of 20%. Red dashed lines represent 20% and 30% prevalence. (TIF) [file pntd.0010456.s003.tif]

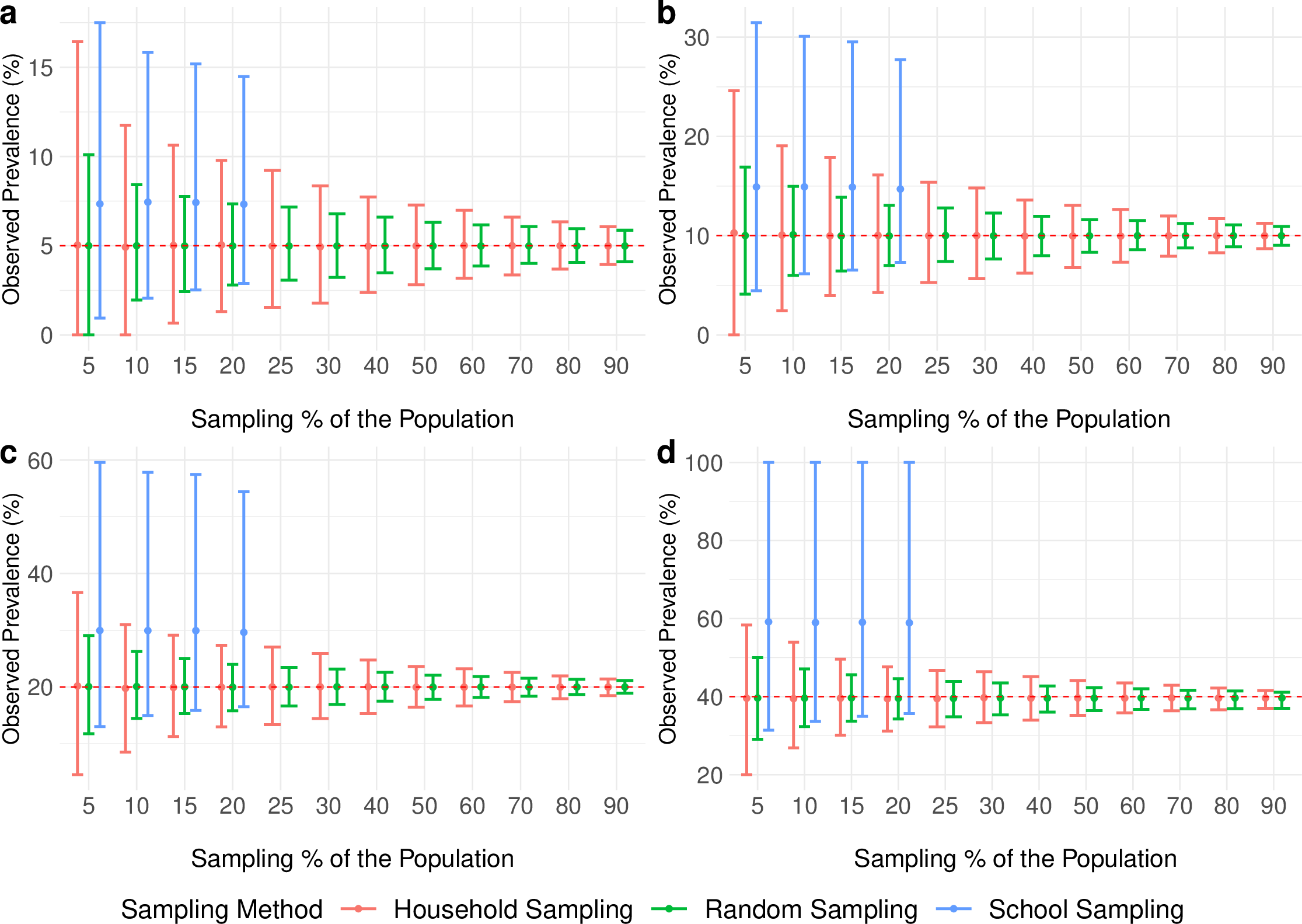

Supplement: S4 Fig — The results (median and 2.5% to 97.5% quantiles) are plotted for four exemplar input prevalence percentages of (a) 5%, (b) 10%, (c) 20%, (d) 40% across all population sizes with a sampling percentage of 20%. Red dashed lines represent the input prevalences. In the school-based sampling strategy the highest sampling percentages could not be achieved due to insufficient population size in the school aged group. (TIF) [file pntd.0010456.s004.tif]

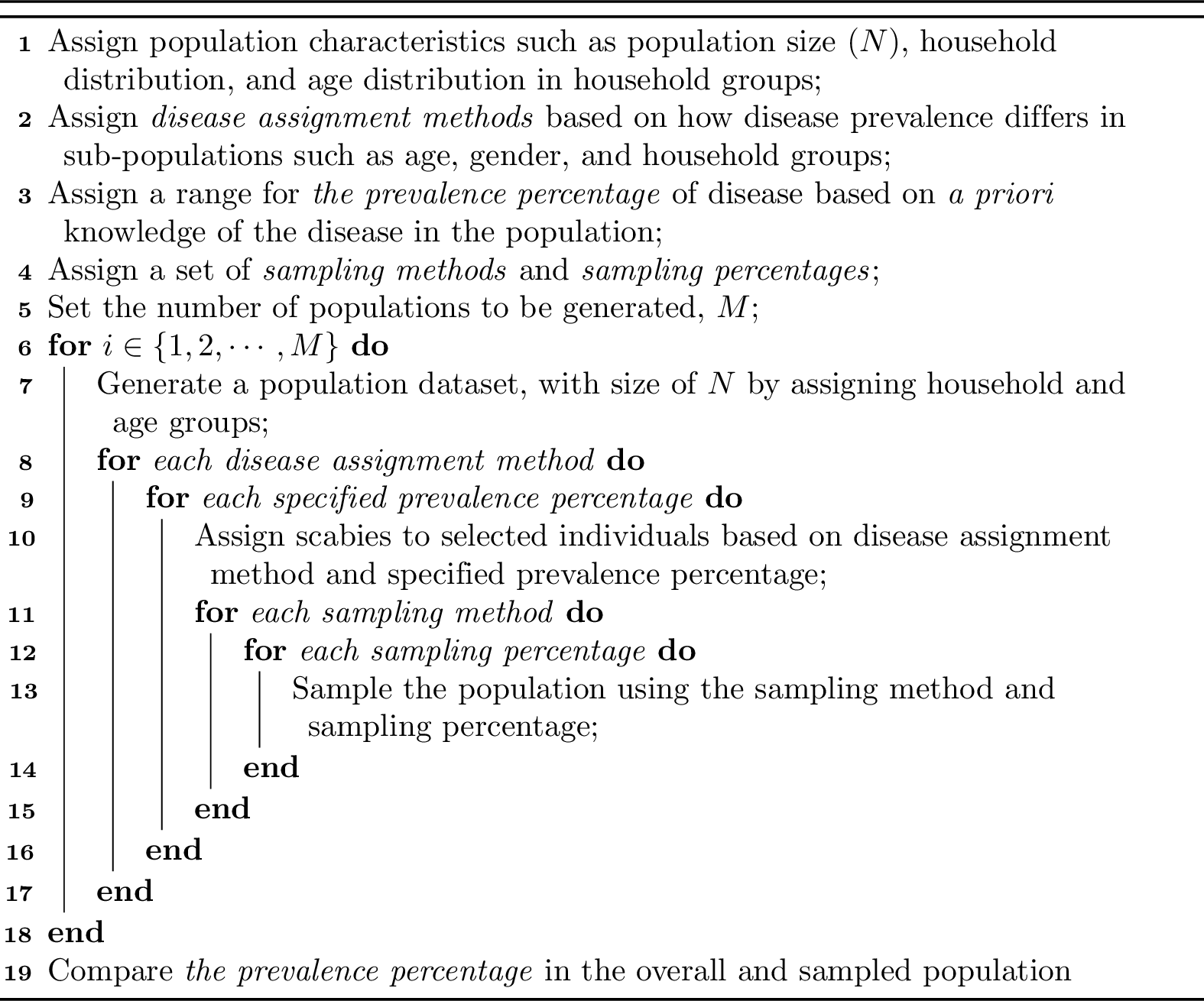

Supplement: S5 Fig — (TIF) [file pntd.0010456.s005.tif]
